# Supplementary material for: Identification of photoperiod-induced specific miRNAs in the adrenal glands of Sunite sheep (Ovis aries)
Source: Front Vet Sci. 2022 Jul 22;9:888207. doi: 10.3389/fvets.2022.888207 (PMC9354845; doi:10.3389/fvets.2022.888207)
Supplement: Supplementary file 1 [file Data_Sheet_1.ZIP › Supplementary file/miRDeep2 file.zip!/miRDeep2 file/Know_Structure/LP42A2/oar-mir-323c.pdf]

miRBase precursor	: oar-mir-323c
Total read count	: 138
oar-miR-323c read count	: 134
remaining reads	: 4

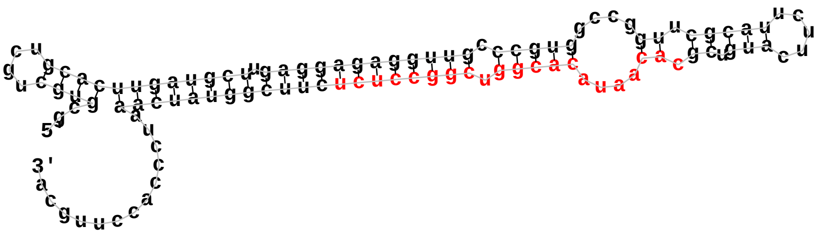

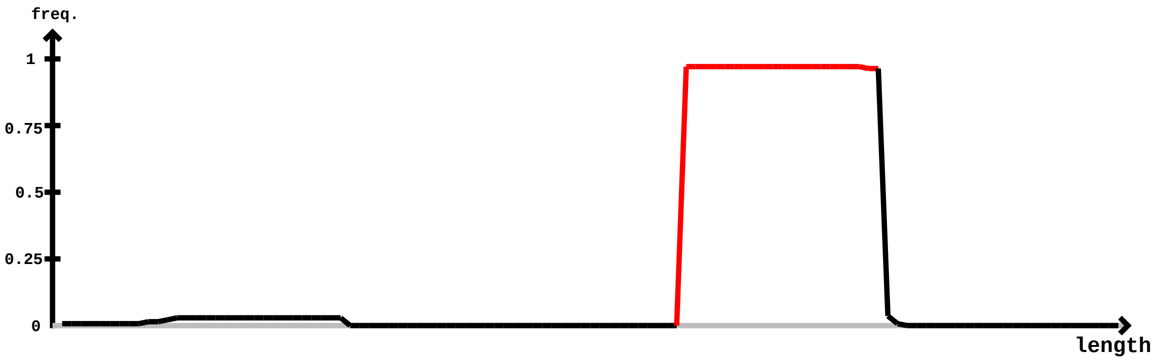


```
                                                                oar-miR-323c
5'- gcgugcugcugcacuugaugcuugaggagagguugcccguggccgguucgcauucuucaugucgcacaauacacggucggccucucuucgguaucaaaucccaccuugca   -3'    exp
    ..((((....))))((((((((.((((((((((((.(((((....((.(((((.....))).)).)).))....))))).))))))))))))))))))))).............    reads      mm          sample
    gcgugcugcugcacuugaugcuugaggag.................................................................................    1          0           seq
    ........cugcacuugaugcuugaggag.................................................................................    1          0           seq
    ..........gcacuugaugcuugaggag.................................................................................    1          0           seq
    ...........cacuugaugcuugaggag.................................................................................    1          0           seq
    ................................................................cacaauacacggucggccu...........................    1          0           seq
    ................................................................cacaauacacggucggccucu.........................    128        0           seq
    ................................................................cacaauacacggucggccucuc........................    4          0           seq
    ................................................................cacaauacacggucggccucucu.......................    1          0           seq
```
